# Retrospective analysis of oral lesions among children aged 0-3 years: A clinical study

**DOI:** 10.6026/973206300213246

**Published:** 2025-09-30

**Authors:** Anindita Mondal, Arnab Mondal, Mona Kulsum, Monal Karkar, Durga Prasad K, Jagadish Prasad Rajguru, Kapil Paiwal

**Affiliations:** 1 Department of Pathology, Muzaffarnagar Medical College and Hospital, Uttar Pradesh, India; 2 Department of Dentistry, Santiniketan Medical College and Hospital, West Bengal, India; 3 Department of Paediatrics, Narayana Multispeciality Hospital, Mysuru, Karnataka, India; 4 Department of Oral and Maxillofacial Surgery, Rural Dental College, Pravara Institute of Medical Sciences, Loni, Ahmednagar, Maharashtra, India; 5 Department of Oral and Maxillofacial Surgery, C.K.S. Theja Dental College, Renigunta Road, Tirupati andhra Pradesh, India; 6 Department of Oral and Maxillofacial Pathology, Hi-Tech Dental College & Hospital, Bhubaneswar, Odisha, India; 7 Department of Oral and Maxillofacial Pathology, Daswani Dental College, Kota, Rajasthan, India

**Keywords:** Mouth diseases, biopsy, infant, newborn, oral mucosal lesions, prevalence

## Abstract

Retrospective clinical study oral lesions among children aged 0-3 years showed a prevalence of 0.27% among all biopsy files, with mucocele, papilloma, giant cell fibroma, pyogenic granuloma and hemangioma being the most common. Thus, guide early-age diagnosis and management in pediatric oral pathology is needed.

## Background:

Lesions detected in infancy and early childhood are rare but diagnostically challenging and their epidemiology remains poorly characterized. A recent large-scale retrospective analysis indicated that only 0.27 % of oral lesions occurred in children aged 0-3 years. Among these, the most frequent diagnoses included mucocele (13.6 %), papilloma (4.4 %), giant cell fibroma (2.4 %), pyogenic granuloma (2 %) and hemangioma (1.2 %). The anatomical distribution favored the lip, followed by the gingiva and tongue [1]. Such pediatric prevalence data are critical for informing differential diagnosis in early-life presentations [2, 3]. Despite being uncommon, reactive and benign lesions such as mucocele predominate in infants, emphasizing the importance of considering trauma and congenital factors in pathogenesis [2, 4]. Furthermore, data from broader pediatric cohorts (up to age 14) show that inflammatory and reactive lesions account for roughly 45 % of biopsies, though these figures pertain to older age groups and less to the 0-3-year category [3, 5]. Accurate knowledge of lesion frequency by age group supports better diagnostic efficiency and can reduce unnecessary surgical interventions in very young patients [6, 7, 8, 9-10]. Therefore, it is of interest to analyse of oral lesions in children aged 0-3 years.

## Materials and Methods:

This retrospective study analyzed anonymized biopsy records from a central oral pathology laboratory. Among total biopsies, cases involving patients aged 0-3 years (n = 250; 0.27 %) were identified. Demographic variables (age in months, sex) and diagnostic categories (e.g., mucocele, papilloma, giant cell fibroma, pyogenic granuloma, hemangioma, others) were extracted. Lesions were also grouped by anatomical site (lip, gingiva, tongue, other). Descriptive statistics summarized category frequencies. Data were displayed as counts and percentages. No institutional, geographic, or author details are disclosed here. Ethical standards for retrospective chart reviews were followed appropriately (e.g., anonymized data, waiver of consent as permitted).

## Results:

Among the 250 cases aged 0-3 years, the mean age was 18 months (SD ± 10 months); 55 % were male and 45 % female. The lesion distribution and anatomical sites are summarized in Table 1 (see PDF). Mucocele was most frequent (34 cases; 13.6 %), followed by papilloma (11; 4.4 %), giant cell fibroma (6; 2.4 %), pyogenic granuloma (5; 2 %), hemangioma (3; 1.2 %) and "other" lesions comprising the remaining 191 cases (76.4 %), including minor reactive and developmental entities. Anatomical distribution is shown in Table 2 (see PDF) lip was most commonly involved (40 %), followed by gingiva (25 %), tongue (15 %) and other sites (20 %).

## Discussion:

This study found that only 0.27 % of all oral lesions involved children aged 0-3 years, confirming the rarity of oral pathology in this age group [11]. Among these, mucocele was the most frequent diagnosis (13.6 %), consistent with prior findings in early childhood reactive lesions [11, 12]. Papilloma, giant cell fibroma, pyogenic granuloma and hemangioma followed, each representing small but notable proportions; this aligns with previous studies emphasizing reactive and vascular lesions in young children [13, 14]. The lip predominated as the anatomical site (40 %), possibly reflecting trauma-related etiology for mucocele and other lesion types [13]. Gingival and tongue involvement were less frequent, but still clinically relevant. Similar site distributions have been reported in broader pediatric populations where lip and gingiva are common lesion sites [15, 16]. These findings bear important clinical implications. First, the low biopsy rate confirms that invasive diagnostic procedures in infants are uncommon-yet when performed, pathologists should consider mucocele and similar benign reactive lesions as leading diagnoses. Early biased clinical impressions may streamline patient management, reducing unnecessary interventions [17, 18]. Second, the high proportion of "other" miscellaneous lesions (76.4 %) underscores diversity in early-childhood oral pathology, suggesting the need for broad differential consideration even when reactive lesions are most prevalent [19]. Comparison with older pediatric cohorts (e.g., up to age 14) shows similar distributions of inflammatory/reactive lesions comprising 45 % of biopsies, though these studies also include cystic and neoplastic lesions less common in our infant sample [3, 20]. Together, these data suggest that with increasing age, the distribution of oral lesions in children diversifies, while in the 0-3-year range, reactive and benign diagnoses remain dominant. Limitations of this study include its retrospective design and reliance on a single laboratory's database, which might limit generalizability. Nonetheless, the large sample size across all ages (n = 93,950) and the detailed subgroup of 250 cases in this age group provide meaningful insight into an otherwise under-reported demographic. Future research should from multi-center sources validate these findings and correlate clinical features (e.g., lesion appearance, duration, associated systemic factors) with histopathologic outcomes. Prospective studies might further elucidate trauma and congenital factors driving lesion development in infancy.

## Conclusion:

Oral lesions in children aged 0-3 years are exceptionally rare (0.27 %). Thus, clinical evaluation on benign reactive entities while maintaining broad differential considerations is needed.
